# Biodegradable materials for surgical management of infective endocarditis: new solution or a dead end street?

**DOI:** 10.1186/1471-2482-14-48

**Published:** 2014-08-03

**Authors:** Patrick O Myers, Mustafa Cikirikcioglu, Afksendiyos Kalangos

**Affiliations:** 1 Cardiovascular Surgery, Geneva University Hospitals & Faculty of Medicine, 4 rue Gabrielle-Perret-Gentil, 1211 Geneva, Switzerland

**Keywords:** Endocarditis, Valve repair, Biodegradable materials

## Abstract

**Background:**

One third of patients with infective endocarditis will require operative intervention. Given the superiority of valve repair over valve replacement in many indications other than endocarditis, there has been increasing interest and an increasing number of reports of excellent results of valve repair in acute infective endocarditis. The theoretically ideal material for valve repair in this setting is non-permanent, “vanishing” material, not at risk of seeding or colonization. The goal of this contribution is to review currently available data on biodegradable materials for valve repair in infective endocarditis.

**Discussion:**

Rigorous electronic and manual literature searches were conducted to identify reports of biodegradable materials for valve repair in infective endocarditis. Articles were identified in electronic database searches of Medline, Embase and the Cochrane Library, using a predetermined search strategy. 49 manuscripts were included in the review. Prosthetic materials needed for valve repair can be summarized into annuloplasty rings to remodel the mitral or tricuspid annulus, and patch materials to replace resected valvar tissue. The commercially available biodegradable annuloplasty ring has shown interesting clinical results in a single-center experience; however further data is required for validation and longer follow-up. Unmodified extra-cellular matrix patches, such as small intestinal submucosa, have had promising initial experimental and clinical results in non-infected valve repair, although in valve repair for endocarditis has been reported in only one patient, and concerns have been raised regarding their mechanical stability in an infected field.

**Summary:**

These evolving biodegradable devices offer the potential for valve repair with degradable materials replaced with autologous tissue, which could further improve the results of valve repair for infective endocarditis. This is an evolving field with promising experimental or initial clinical results, however long-term outcomes are lacking and further data is necessary to validate this theoretically interesting approach to infective endocarditis.

## Background

Infective endocarditis is a serious disease with an incidence of 30 to 100 per million patient-years [1-3]. Mortality is high: more than a third of patients will die within the first year of diagnosis [4,5]. The role of surgery in the treatment of infective endocarditis has been expanding, and current guidelines advocate surgical management for complicated infective endocarditis [6,7]; approximately one third of patients with infective endocarditis will require operative intervention [8]. Early surgery is strongly indicated for patients with infective endocarditis and congestive heart failure, large left-sided vegetations (>10 mm) at high risk of embolism, persistent or locally uncontrolled infection (abscess, false aneurysm, fistula or enlarging vegetation), varying between a recommendation of class I to IIa and a level of evidence of B to C [5].

Operative principles for infective endocarditis include complete debridement of infected tissues, drainage of abscess cavities followed by restoration of anatomic relationships, such as ventriculo-aortic or atrioventricular continuity [9]. Several surgical options exist to restore a competent valve depending on the extent of valve destruction by the infectious process, ranging from valve repair to valve replacement. Valve replacement with an off-the shelf prosthetic valve in the setting of active infection can lead to recurrent infection, estimated between 2% to 9% [9,10]. Infection-resistant prosthetic valves have been developed, however their results are poor and no devices are currently approved for clinical use. The multi-center prospective randomized Artificial Valve Endocarditis Reduction Trial (AVERT), using a prosthetic valve made with a silver Silzone-coated sewing cuff, was stopped before completion due to a higher rate of major paravalvular leaks requiring reoperation [11]. Other modalities, such as biodegradable matrices that secrete antibacterial proteins [12], are still at the stage of basic research and have yet to be tested in clinical settings. Homografts have been advanced as an ideal substitute in the setting of active endocarditis due to their biological nature and absence of foreign woven material, although they remain susceptible to recurrent infection [13] and complicate the initial and subsequent valve replacements.

Given the superiority of valve repair over valve replacement in many indications [14,15] other than endocarditis, there has been increasing interest and an increasing number of reports of excellent results of valve repair in acute infective endocarditis [16].

## Discussion

### Rationale for Biodegradable Materials in Infective Endocarditis

Monofilament suture has been shown to be less susceptible to colonization in *in vitro* and *in vivo* laboratory studies compared to braided multifilament suture [17-20]. However, in the setting of an infected surgical field which requires placement of foreign material to perform the planned operation, the theoretically ideal material is non-permanent, “vanishing” material [21], dissolving and to be replaced by autologous tissue. The prosthetic materials needed for valve repair can be summarized into annuloplasty rings to remodel the mitral or tricuspid annulus, and patch materials to replace resected valvar tissue.

With the use of adapted perioperative antibiotics and complete surgical debridement, the rate of recurrent endocarditis is, as mentioned previously, quite low both in valve repair and replacement. This implies that any study looking at these technologies, beyond the “proof of concept” phase, will need a large number of patients and rigorous follow-up to have sufficient power to see if these new biodegradable materials can do better than conventional techniques.

### Biodegradable Annuloplasty

It is tempting to avoid an annuloplasty ring when fixing limited leaflet destruction from mitral or tricuspid valve infective endocarditis [22], under the assumption that the underlying mechanism is acute regurgitation and usually doesn’t involve annular dilatation. Annuloplasty plays an important role in valve reconstruction, particularly if a significant infected leaflet segment must be resected, to relieve tension on the repaired leaflets and ensure long-term stability of the repair. Traditional annuloplasty rings and bands, predominantly made of polyester mesh, are susceptible to seeding and infection. Ciprofloxacin-coated polyester annuloplasty ring mesh was shown to confer infection resistance in a subcutaneous animal implantation model [23], however these devices haven’t been reported in clinical use to date.

Temporary annuloplasty with a biodegradable device replaced with autologous fibrous tissue would theoretically reduce the risk of infection recurrence and durably remodel the annulus. Duran et *al*. reported the results of DeVega annuloplasty using 2–0 polydioxanone suture in an experimental sheep model [24], as well as their clinical results in 73 patients with functional tricuspid regurgitation [25], showing that this provided temporary “vanishing” annuloplasty that stabilizes the annulus for 4 months. There has been no further follow-up data from this patient cohort, and no similar reports beyond functional tricuspid regurgitation.

**Figure 1:**
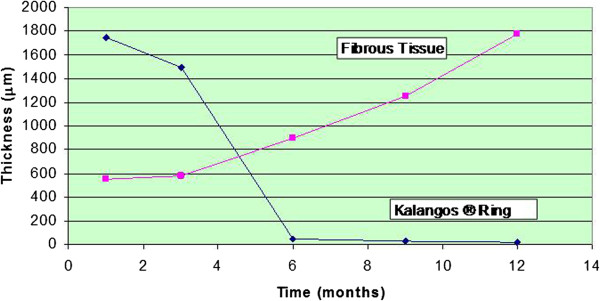
**Degradation and fibrous tissue deposition after biodegradable annuloplasty implantation (reproduced with permission from Kalangos et al**[26]**).**

The Bioring Kalangos® annuloplasty ring (formerly Bioring SA, Lonay, Switzerland) was developed to extend the annular stabilization beyond this time period, while remaining biodegradable. It is a curved “C” segment of poly-1,4-dioxanone polymer located on a non-degradable polyvinyl monofilament suture equipped with a stainless steel needle at each extremity. This ring is inserted subendocardially directly into the mitral or tricuspid annulus, away from blood contact. In a juvenile pig model [26], it was shown to dissolve in 6 months and be replaced at 12 months by autologous fibrous tissue (see Figure 1). The close proximity of important structures to the posterior mitral annulus, such as the circumflex coronary artery and the great cardiac vein, has raised questions regarding the safety of implanting the ring into the native annulus. This issue was addressed by Cikirikcioglu et *al*. in a cadaveric study, which showed that the distances between the ring and the circumflex coronary artery was 7.2 ± 2.7 mm, 11.0 ± 2.4 mm, and 10.7 ± 3.8 mm at the level of anterolateral commissure, the mid posterior annulus, and the posteromedial commissure, respectively [27].

Clinical results have been reported in children with rheumatic mitral regurgitation [28], congenital mitral [29,30] and tricuspid [31] regurgitation. The results have been positive so far, with evidence of durable stabilization of the mitral or tricuspid annulus while allowing for growth in this pediatric patient population, as evidenced by stable annular diameter Z-scores. Panos et *al*. also reported the ease of implantation of this ring in minimally invasive thoracoscopic or robotic mitral and tricuspid valve repair [32]. Finally, Pektok et *al*. reported the histological findings 21 months after implantation in an adult patient with ischemic mitral regurgitation, confirming that the ring is degraded and replaced by dense autologous fibrous tissue along the posterior mitral annulus [33].

The infection resistance of the biodegradable annuloplasty ring was tested in an *in vivo* rat animal model study, compared to the Carpentier-Edwards (Edwards Lifesciences Corp, Irvine, CA, USA) Physio II ring (M. Cikirikcioglu, personal communication, presented at AATS mitral conclave 2013, poster E57: “infection resistance testing of biodegradable annuloplasty ring in a rat subcutaneous implantation model”). In this study, segments of annuloplasty ring were implanted subcutaneously and topically inoculated with 2×10^7^ colony forming units (CFU) of *Staphylococcus aureus*. Cultures of explanted rings were more frequently positive in conventional rings (11/16) than the biodegradable ring (2/16, *P* = 0.003), and the amount of growing bacteria was also significantly increased in conventional rings (7175 ± 5936 CFU/ml vs. 181 ± 130 CFU/ml in the biodegradable ring, *P* = 0.0005).

Clinical use of the biodegradable ring has been reported for valve repair in infective endocarditis by Kazaz et *al*. [34] and by our group [35,36]. From 2004 to 2009, we implanted the ring in 17 patients with acute infective endocarditis, 13 in the mitral, 3 in the tricuspid and 1 in both valves [35]. There were 3 early deaths, and no late evidence of endocarditis recurrence, valve dysfunction, reoperations or deaths at a mean follow-up of 30 months. We updated this report with our experience in 8 children with infective endocarditis [36]. There were no early or late deaths, reoperations or evidence of endocarditis recurrence at a mean follow-up of 56 months.

These different studies are limited for the most part by the fact that most data originated from our group (and are currently being validated by more widespread use of these devices) and a follow-up that extends to a maximum of 9 years now. This data represents little more than a “proof of concept”, providing data on our initial favorable experience, and mandates larger multi-center prospective randomized studies to validate these initial findings made in selected patients in a the hands of dedicated team.

### Biodegradable Patches

Surgical management of infective endocarditis entails debridement of all infected tissue, which may include leaflets, the annulus or structures beyond the annulus (such as the atrioventricular groove or the ventriculo-arterial continuity), which require reconstruction. There is relatively little data on leaflet patch reconstruction in infective endocarditis, beyond a few limited case reports or series. More generally in leaflet repair, all available patch materials have disappointing mid- and long-term results. Autologous fresh pericardium retracts with time, while glutaraldehyde-fixed pericardium calcifies [37]. Expanded polytetrafluoroethylene has been proposed as a leaflet repair patch material [38], however mid- and long-term follow-up data is still pending and this material may be at risk of calcification [39] or late fracture [40]. Each of these materials are permanent, non-degradable and at risk of seeding and the cause of recurrent infection.

Given the poor long-term results of leaflet patch augmentation overall (and not just in the setting of infective endocarditis), new patch materials are being developed to realize the dream of *in vivo* valvulogenesis, using scaffolds intended to be colonized and replaced by autologous tissue. Several synthetic scaffolds have been proposed *in vitro* and in animal models, such as polydiaxonone [41], polyglycolic acid, poly–L-lactic acid [42] or polyurethane. Their clinical application as a biodegradable patch for valve repair remains to be reported.

**Figure 2:**
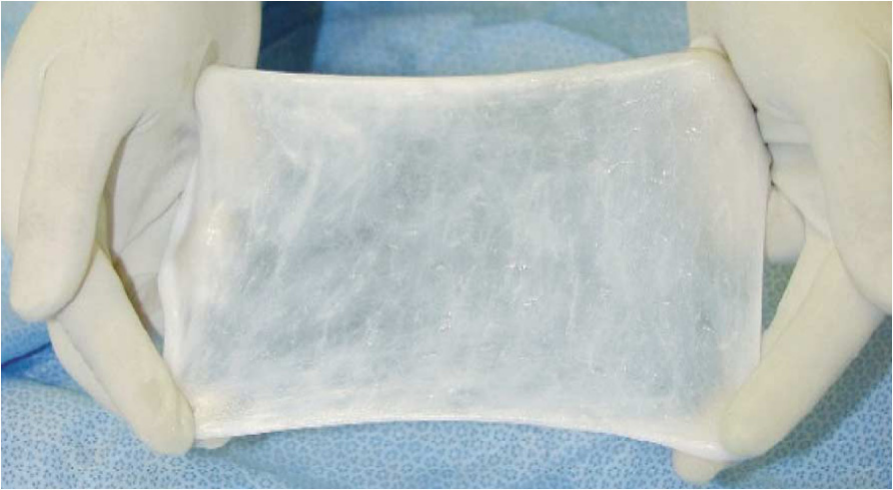
**ECM harvested from porcine urinary bladder.** This thin (60 uM) sheet of ECM is entirely free of any cellular component, has a multidirectional tensile strength of approximately 40 N, and has not been chemically cross linked or modified from its native structure. Reproduced with permission from Badylak et al [43].

Biological scaffolds have also been developed, predominantly using extracellular matrix (ECM). The structural and functional components of ECM are transient, due to the rapid rate of degradation of ECM scaffolds in vivo [43]; they could be considered as temporary controlled release vehicles for naturally occurring growth factors. The characteristic of the intact ECM that distinguishes it from other scaffold materials is its diversity of structural proteins and associated bioactive molecules and their unique spatial distribution. ECM can be harvested for use as a therapeutic scaffold from the dermis, submucosa of the small intestine and urinary bladder, pericardium, basement membrane and stroma of the decellularized liver, and the decellularized Achilles tendon [43] (see Figure 2). Although there is abundant literature on modified ECM as a scaffold (e.g. in biological prosthetic heart valves or glutaraldehyde-treated pericardium), the data is newer on ECM that hasn’t been modified, except for decellularlization and sterilization. As reviewed in detail by Badylak [43], ECM scaffolds that remain essentially unchanged from native ECM elicit a host response that promotes cell infiltration and rapid scaffold degradation, deposition of host derived neomatrix and constructive tissue remodeling with minimal scar tissue; this represents a fundamentally different scaffold material than ECM that has been chemically or otherwise modified.

CorMatrix© is a commercially available ECM scaffold made of non-modified small intestinal submucosa. Initially approved for pericardial replacement, its use has been expanded as a patch for intracardiac [44] and vascular reconstruction [45]. Its application in heart valve repair is relatively new, and there is a paucity of data available. In a retrospective study of 25 children with congenital mitral or tricuspid lesions who underwent leaflet patch augmentation with CorMatrix©, the reoperation rate was similar to matched controls who had leaflet patch augmentation with glutaraldehyde-treated pericardium at 12 months follow-up, although the mechanism of failure tended to differ, with more patch retractions in the pericardium group [46]. None of these patients were operated on in the setting of acute infective endocarditis. There is only one report of CorMatrix© use in infective endocarditis [47], reporting posterior mitral leaflet augmentation with CorMatrix© for mitral valve *S. viridans* endocarditis in one patient, and previously repaired ventricular septal defect patch replacement with CorMatrix© for *S. aureus* septal patch endocarditis and aortic root abscess. Both these patients had favorable outcomes, with local (gentamycin fleece left in situ around an aortic root graft) and systemic adapted antibiotics, although follow-up was limited to 34 days and 3 months, respectively in each patient.

**Figure 3:**
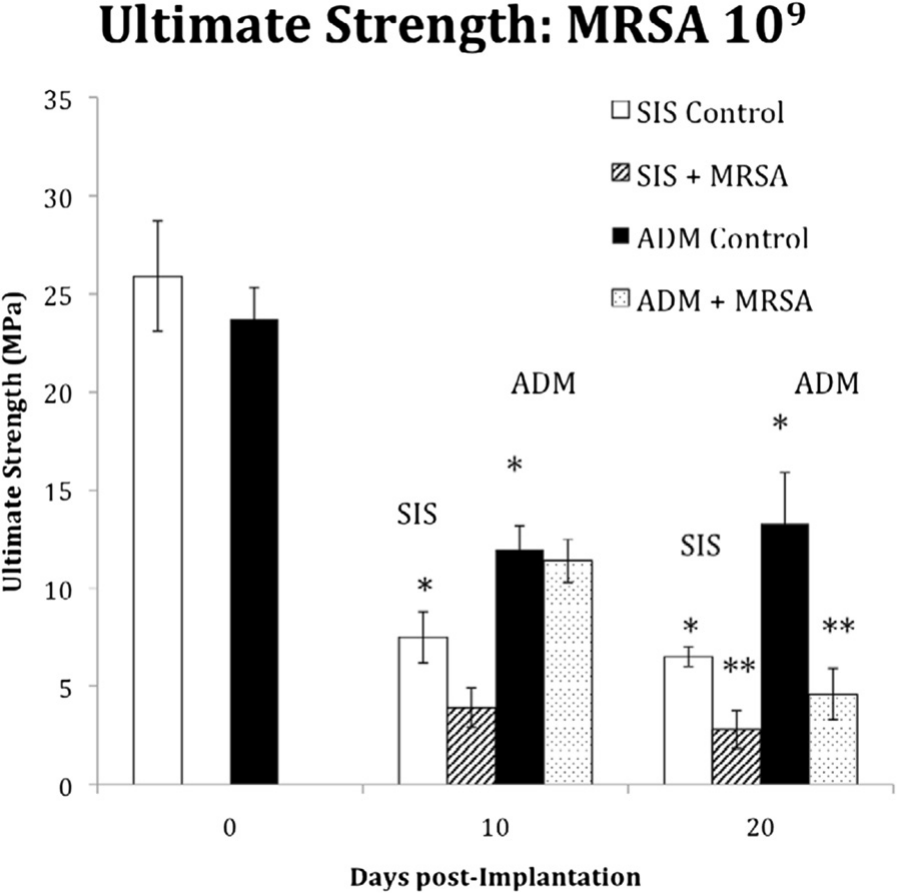
**Ultimate strength of extracellular matrix scaffolds subjected to infection.** Ultimate strength (MPa) of small intestinal submucosa (SIS) and acellular human dermis (ADM) in response to inoculation with MRSA. White bars and black bars represent the control (non-inoculated) values for the 2 biologic meshes. Both materials exhibited the greatest reduction in ultimate strength at 20 days post inoculation. *Indicates a statistically significant difference between control groups. **Indicates a statistically significant difference between inoculated and control groups. Reproduced with permission from Bellows et al. [49].

Although these biodegradable materials have promising initial experimental and clinical results in non-infected valve repair, as well as theoretical advantages in an infected surgical field, there are limited reports of using ECM scaffolds for valve repair in infective endocarditis. In comparison, abdominal wall hernia repair using ECM scaffolds in infected settings have shown reasonable results at most, with simplified wound care and lower rate of reoperation for matrix removal compared to non-degradable materials [48], although the rate of hernia recurrence remained as high a 30%. This last point raises the question of the adequacy of a biodegradable patch material for leaflet reconstruction during active infection, as the patch will be subjected to very high pressures while it is being remodeled. In an *in vitro* comparison of the biomechanical properties of patch materials after experimental infection with MRSA [49], ECM scaffolds showed a significant decrease in the ultimate tensile strength and modulus of elasticity compared to non-infected controls (see Figure 3). Taking into account both the indirect clinical observations cited above, and this *in vitro* experimental data, serious concerns should be raised on the use of ECM scaffolds in infective endocarditis, and thorough *in vivo* animal experiments are required to evaluate the biomechanical properties of these patch materials in this specific setting before clinical use.

### Summary

These evolving biodegradable devices, from annuloplasty rings to patch materials, offer the potential for valve repair with degradable materials replaced with autologous tissue. This is a perfect example of translational medicine, going from *in vivo* animal models investigating biodegradation and resistance to infection, to clinical application in the hopes that these properties could further improve the results of valve repair for infective endocarditis. This is an evolving field with promising experimental or initial clinical results, however long-term results are lacking and further data is necessary to validate this theoretically interesting approach to infective endocarditis. Furthermore, given the low rate of recurrent endocarditis using non-degradable materials, any study looking at these technologies, beyond the “proof of concept” phase we are emerging from, will need a large number of patients and rigorous follow-up to have sufficient power to see if these new biodegradable materials can do better than conventional techniques.

## Abbreviations

AATS: American association of thoracic surgeons; ADM: Acellular human dermis; AVERT: Artificial Valve Endocarditis Reduction Trial; CFU: colony forming unit; ECM: extracellular matrix; ml: millilitre; MPa: megapascal (modulus of elasticity); MRSA: methycilin-resistant staphylococcus aureus; SIS: small intestinal submucosa.

## Competing interests

AK was a consultant for Bioring SA, Lonay, Switzerland. None of the other authors declare any conflict of interest.

## Authors’ contributions

P.O.M conceived the review, performed literature searches and drafted the debate. M.C. helped conceive the review, performed literature searches and critically reviewed the manuscript. A.K. helped conceive the review, participated in its coordination and critically reviewed the manuscript. All authors read and approved the final manuscript.

## Pre-publication history

The pre-publication history for this paper can be accessed here:

http://www.biomedcentral.com/1471-2482/14/48/prepub
